# Supplementary material for: Getting More Out of Biomedical Documents with GATE's Full Lifecycle Open Source Text Analytics
Source: PLoS Comput Biol. 2013 Feb 7;9(2):e1002854. doi: 10.1371/journal.pcbi.1002854 (PMC3567135; doi:10.1371/journal.pcbi.1002854)
Supplement: Dataset S1 — GATE software. Dataset S1 bundled with this paper contains a distribution of GATE (or see http://gate.ac.uk/download/). (TGZ) [file pcbi.1002854.s001.tgz › plos-gate/plugins/Gazetteer_LKB/doc/semantic_encrichment_pr.html]

The Large KB gazetteer - Semantic Enrichment PR


---

Last Published: 2009-10-17

---

##### Large KB Gazetteer

- Overview
- Usage
- Q & A
- GATE Compatibility
- GATE Gazetteer Feature Comparison
- **Semantic Enrichment PR**
- Samples

##### Developer Documentation

- Dictionary Lifecycle

The Semantic Enrichment PR allows adding new data to semantic annotations by querying external RDF (Linked Data) repositories. It is a companion to the large KB gazetteer that showcases the usefulness of using Linked Data URI as identifiers.

Here a semantic annotation is an annotation that is linked to an RDF entity by having the URI of the entity in the "inst" feature of the annotation. For all such annotation of a given type, this PR runs a SPARQL query against the defined repository and puts a comma-separated list of the values mentioned in the query output in the "connections" feature of the same annotation.

There is a sample pipeline that features the Semantic Enrichment PR.

## Parameters

- inputASName - The annotation set, which annotation will be processed.
- server - The URL of the Sesame 2 HTTP repository. Support for generic SPARQL endpoints can be implemented if required.
- repositoryId - The ID of the Sesame repository.
- annotationTypes - A list of types of annotation tha twill be processed.
- query - A SPARQL query pattern. The query will be processed like this - String.format(query, uriFromAnnotation), so you can use parameters like %s or %1$s.
- deleteOnNoRelations - Whether we want to delete the annotation that weren't encriched. Helps to clean up the input annotations.

---

©
2009
Ontotext AD

---
